# Personalised Exercise Rehabilitation FOR people with Multiple long-term conditions (PERFORM): findings from a process evaluation of a randomised feasibility study

**DOI:** 10.1136/bmjopen-2025-100199

**Published:** 2025-09-17

**Authors:** Sophie Eleanor Brown, Sharon Anne Anne Simpson, Colin Greaves, Paulina Daw, Sarah Gerard Dean, Rachael A Evans, Tom M Withers, Zahira Ahmed, Shaun Barber, Gwen Barwell, Patrick Joseph Doherty, Nikki Gardiner, Tracy Ibbotson, Bhautesh Jani, Kate Jolly, Frances Mair, James R Manifield, Emma McIntosh, Daniel Miller, Paula Ormandy, Susan Smith, Ioannis Vogiatzis, Ghazala Waheed, Rod Taylor, Sally J Singh, on behalf of the PERFORM research team, Sayem Ahmed

**Affiliations:** 1 School of Health and Wellbeing, University of Glasgow, Glasgow, UK; 2 School of Sport, Exercise and Rehabilitation Sciences, University of Birmingham, Birmingham, UK; 3 Medical School, University of Exeter, Exeter, UK; 4 Respiratory Sciences, University of Leicester, Leicester, UK; 5 Centre for Exercise and Rehabilitation Science, NIHR Leicester Biomedical Research Centre Respiratory Diseases, Leicester, UK; 6 Leicester Clinical Trials Unit, University of Leicester, Leicester, UK; 7 Health Sciences, University of York, York, UK; 8 University Hospitals of Leicester NHS Trust, Leicester, UK; 9 General Practice and Primary Care, School of Health and Wellbeing, University of Glasgow, Glasgow, UK; 10 Department of Applied Health Research, University of Birmingham, Birmingham, UK; 11 Health Economics and Health Technology Assessment (HEHTA), University of Glasgow, Glasgow, UK; 12 University of Leicester, Leicester, UK; 13 School of Health and Society, University of Salford, Salford, UK; 14 School of Medicine, Trinity College Dublin, Dublin, Ireland; 15 Department of Sport, Exercise and Rehabilitation, Northumbria University, Newcastle upon Tyne, UK; 16 Robertson Centre for Biostatistics, University of Glasgow, Glasgow, UK

**Keywords:** Multimorbidity, Exercise, Self-Management, REHABILITATION MEDICINE

## Abstract

**Objective:**

The number of people living with multiple long-term conditions (MLTCs or ‘multimorbidity’) is growing. Evidence indicates that exercise-based rehabilitation can improve health-related quality of life and reduce hospital admissions for a number of single long-term conditions. However, it is increasingly recognised that such condition-focused rehabilitation programmes do not meet the needs of people living with MLTCs. The aims for this study were to (1) evaluate the acceptability and feasibility of the newly developed Personalised Exercise Rehabilitation FOR people with Multiple long-term conditions (PERFORM) intervention; (2) assess the feasibility of study methods to inform progression to a definitive randomised controlled trial (RCT) and (3) refine our intervention programme theory.

**Design:**

Semi-structured qualitative interviews were conducted with patients receiving and healthcare practitioners delivering the PERFORM intervention, to seek their experiences of the intervention and taking part in the study. Interviews were analysed thematically, informed by Normalisation Process Theory and the programme theory.

**Setting:**

Three UK sites (two acute hospital settings, one community-based healthcare setting).

**Participants:**

18 of the 60 PERFORM participants and 6 healthcare professionals were interviewed.

**Intervention:**

The intervention consisted of 8 weeks of supervised group-based exercise rehabilitation and structured self-care symptom-based support.

**Results:**

All participants and staff interviewed found PERFORM useful for physical and mental well-being and noted positive impacts of participation, although some specific modifications to the intervention delivery and training and study methods were identified. Scheduling, staffing and space limitations were barriers that must be considered for future evaluation and implementation. Key intervention mechanisms identified were social support, patient education, building routines and habits, as well as support from healthcare professionals.

**Conclusions:**

We found the PERFORM intervention to be acceptable and feasible, with the potential to improve the health and well-being of people with MLTCs. The findings of the process evaluation inform the future delivery of the PERFORM intervention and the design of our planned full RCT. A definitive trial is needed to assess the clinical and cost-effectiveness.

**Trial registration number:**

ISRCTN68786622.

**STRENGTHS AND LIMITATIONS OF THIS STUDY**

- This study evaluated an innovative exercise programme for people with multiple long-term conditions.
- Normalisation Process Theory and the programme theory provided a strong framework to uncover the work required to take part in or deliver the intervention.
- Sample diversity was limited by the number of participants who consented to be contacted and were available for interview.
- Interviewing managerial staff who were not directly responsible for intervention deliver may have produced a more thorough understanding of the potential challenges facing sites.

## Introduction

### Multimorbidity and exercise rehabilitation

 A growing number of individuals worldwide experience living with multiple long-term conditions (MLTCs, or ‘multimorbidity’), the presence of at least two long-term conditions. It is currently estimated that one in three of the world’s population live with MLTCs,^1^ making it a significant public health concern. The presence of MLTCs is associated with functional decline, reduced health-related quality of life (HRQoL), higher risk of hospitalisation and mortality, and increased healthcare costs.^1^

The latest Cochrane systematic review did not identify any current effective interventions specifically designed to support people with MLTCs to manage their conditions.^2^ Addressing this unmet rehabilitation need is a global priority.^3^ There is extensive evidence illustrating the positive effects of structured exercise-based rehabilitation for many single LTCs, including improved HRQoL and functional capacity.^4^,^10^ Exercise-based rehabilitation interventions typically involve a period of supervised exercise alongside self-management support and symptom management.^5^,^811 12^ However, existing exercise-based rehabilitation services are typically targeted towards single conditions, most commonly cardiac or pulmonary condition LTCs.^13^,^15^ New models of delivery are therefore needed to support the more complex needs of people with MLTCs.

As part of the Personalised Exercise Rehabilitation FOR people with Multiple long-term conditions (PERFORM) programme of research,^16^,^18^ we developed an exercise-based rehabilitation and self-care support programme to improve HRQoL in people with MLTCs. We conducted a randomised feasibility study in three UK sites to test the feasibility and acceptability of the PERFORM intervention and the study methods.^17^ This article presents the qualitative results of a process evaluation conducted as part of this wider study. Full feasibility trial results are reported elsewhere.

### The PERFORM intervention and programme theory

PERFORM is a novel exercise-based rehabilitation programme developed for people with MLTCs. It was co-designed with patients, service commissioners and service providers using stakeholder workshops, evidence synthesis and identification of supportive behaviour change techniques to facilitate patient engagement.

The programme runs for 8 weeks and consists of group-based exercise and health and well-being self-management sessions supervised by healthcare professionals. Sessions were twice weekly for 6 weeks, then weekly for 2 weeks. Each session includes 1 hour of supervised exercise and 1 hour of education and discussion on various self-care topics relevant to people with MLTCs, such as diet and stress management. Exercise is progressive and prescribed individually, focusing on aerobic fitness and resistance training, and participants additionally undertake tailored home exercise activities. Participants complete a ‘progress tracker’ diary to record their goals, exercise and any changes in self-care behaviours. Feasibility study participants were offered group-based maintenance sessions at 4 and 6 months following programme completion to review progress and provide further support. Health Care Practitioners (HCPs) responsible for the delivery of the PERFORM intervention were provided with one 6-hour, in-person group training session covering delivery of the exercise rehabilitation programme and the health and well-being education sessions.

PERFORM uses evidence-based behaviour change techniques to promote change in the target exercise, lifestyle and self-management behaviours (see logic model, online supplemental file 1). The theoretical underpinnings/key principles of the PERFORM intervention include

- Building autonomy and empathy (using person-centred counselling).^19^
- Tailoring exercise and self-care support to individual needs.
- Self-regulation (building self-efficacy/competence via a cycle of realistic action-planning, self-monitoring, reviewing progress and problem-solving).^20^
- Building a functional ‘illness model’ (as defined in Leventhal’s Common Sense Model of self-regulation)^21^ to help patients see how self-care behaviours will affect their physical and mental well-being.
- Facilitating the process of psychological adaptation to living with chronic conditions (via acceptance-based approaches/finding a new normal).^22^
- Engaging/building social support.^23^
- Cognitive behavioural strategies^24^ and mindfulness-based approaches^25^ to manage the psychological consequences of living with MLTCs (such as stress, anxiety, frustration and low mood).

### Study aims

To assess the feasibility and acceptability of the PERFORM intervention exploring barriers and facilitators to uptake and engagement from both participant and healthcare provider perspectives; (2) to assess the feasibility of the study methods and (3) to refine the PERFORM intervention and programme theory to inform progression to a full randomised controlled trial (RCT). This article reports only on feasibility and acceptability as examined via qualitative interviews. ^17^

## Methods

### Study design

Details of the feasibility study overall design and methods are reported in our published protocol paper.^16^ In summary, we used a parallel two-group randomised trial design with individual 2:1 allocation to the PERFORM exercise-based intervention plus usual care (intervention group) or usual care alone (control group) and nested mixed-methods process and economic evaluation. The study was conducted across three UK sites (Leicester, Newcastle and Northampton) with expertise in exercise rehabilitation-based services for LTCs and recruited a total of 60 people with MLTCs, defined as two or more long-term conditions, with at least one having evidence of the beneficial effect of exercise.^26^

Feasibility and participant outcome data were collected at baseline (pre-randomisation) and 3 months post randomisation. The participant outcomes, quantitative process evaluation data (PERFORM intervention attendance and fidelity) and cost data are reported elsewhere.^17^ The remainder of this article focuses on the qualitative process evaluation methods and findings.

### Study procedures

We conducted in-depth interviews with (1) patients participating in the intervention and (2) HCPs involved in delivery. Patients from all three study sites who consented to being contacted about interviews were purposively sampled across age, gender, socioeconomic status and conditions. All HCPs involved in intervention delivery were invited to participate in interviews. Contact details for participants and HCPs were shared securely by the Leicester Clinical Trials Unit with SEB, who contacted participants via email or phone to discuss taking part in an interview. Participants were emailed or posted the study information sheet and consent form. Consent forms were completed verbally prior to interviews and signed electronically by SEB on behalf of participants with their permission. The consent process was also audio recorded.

### Study sites

The study was conducted across three sites in England with expertise in delivering exercise rehabilitation, including two National Health Service (NHS) city hospital sites and one community-based rehabilitation service that operates as a charity. All sites had existing exercise rehabilitation services. At the NHS sites, separate rehabilitation services were already provided for cardiopulmonary conditions, musculoskeletal (MSK) and neurological conditions. The community site service offered a single service for patients with any conditions that may benefit from exercise rehabilitation but was not tailored specifically towards those managing MLTCs.

### Theoretical approach

This interview schedules (onlinesupplemental files 2^3^) and analyses for the qualitative component of the process evaluation were informed using the intervention programme theory and Normalisation Process Theory (NPT)^27^ to support understanding of the mechanisms involved in implementing the PERFORM programme. NPT is an implementation theory that has been used effectively in feasibility studies and process evaluations to explore the processes required to implement and integrate complex interventions, including barriers and facilitators to successful implementation.^28^ It can be used to capture influences on implementation at the levels of systems, organisations, communities and individuals.^29^ Four key constructs are used to describe the work required of health professionals and patients to ‘normalise’ an intervention as part of routine practice: coherence (making sense of the intervention), cognitive participation (how individuals ‘buy in’ to the intervention), collective action (the work needed to implement the intervention) and reflexive monitoring (appraising the intervention).^27^

### Data collection and analysis

One-to-one semi-structured interviews were conducted by SEB, a female postdoctoral researcher with experience of conducting and analysing qualitative interviews in a healthcare research context. Interviews were completed via videoconferencing or phone calls after intervention completion for patients and at least 2 months from baseline for HCPs. Only the researcher and participants were present at interviews. Interview schedules (see online supplemental appendices 3 and 4) were piloted with the patient advisory group (PAG) prior to data collection. Patient interviews addressed participants’ experiences of taking part in the PERFORM intervention, including, for example, motivation to attend, barriers and facilitators to engagement, and mechanisms of change. Follow-up interviews were also conducted with a subsample of patient participants following the maintenance sessions at 3 and 6 months to examine the usefulness of those sessions. HCP interviews explored staff experiences of facilitating the intervention, including barriers and facilitators of delivering the PERFORM intervention, the feasibility of implementing it and the perceived impact on patients.

Interviews were audio recorded and transcribed verbatim and de-identified. The interviews were analysed thematically in NVivo using the four core NPT domains (coherence, cognitive participation, collective action reflexive monitoring) and the programme theory (eg, mechanisms of change, maintaining change). Deductive coding was used to map codes onto the NPT domains, and inductive coding was used to identify additional relevant themes outside those domains. Analysis involved a process of familiarisation, preliminary coding of five interviews, development of an initial coding framework, double coding and checking, coding of the remaining interviews and continual refinement of the coding framework. Following preliminary coding by one of the research team (SEB), a second researcher (TMW) double coded 20% of the interviews against the draft coding framework. Disagreements were resolved through discussion and clarification.

### Patient and public involvement

The PERFORM intervention has an established PAG consisting of 13 lay patient members with lived experience of MLTCs and/or participation in cardiorespiratory rehabilitation, and caregivers, representing people from deprived areas and ethnic minority groups. The process evaluation design and interview schedules were reviewed and approved by the PAG prior to data collection.

## Results

### Study sites and participants

The study included two NHS city hospital sites and one community-based rehabilitation service that operates as a charity. All sites had existing exercise rehabilitation services. At the NHS sites, separate services were provided for cardiopulmonary conditions, MSK and neurological conditions. The community site service offered a single service for patients with any conditions that may benefit from exercise rehabilitation.

Of the 40 patients allocated to the PERFORM intervention group, 38 consented to be contacted. Of these, we interviewed 18 participants across three sites (S1=8, S2=6 and S3=4). Reasons for not taking part included no response (no response or unable to contact using details provided) (n=12), illness (n=3) and not interested in participating (n=5). Follow-up interviews to discuss maintenance sessions were carried out with six of the same participants, drawn from those who attended at least one maintenance session and had consented to being contacted (S1=2, S2=3 and S3=1). The six HCPs (two per site) who were responsible for delivering the PERFORM intervention were also interviewed. Interviews lasted between 30 and 75 min. Patient participants experienced a range of conditions and symptoms but most commonly cardiopulmonary and MSK. Most lived in or around cities near to the sites and drove to sessions or were driven, with some using public transport. All HCPs were physiotherapists with cardiopulmonary and exercise rehabilitation experience, and all sites offered some form of cardiopulmonary, MSK and neurological exercise rehabilitation. Patient participant demographic characteristics are listed below:

*Gender:* female (n=8) and male (n=10).
*Age:* 40–49 years (n=3), 50–59 years (n=4), 60–69 years (n=7) and 70–79 years (n=3).
*Work:* retired (n=11), unable to work due to ill health (n=4) and full-time work (n=3).
*Education:* secondary school (n=7), college (n=3), university (n=6) and unknown (n=2).
*Number of conditions:* 2–3 (n=5), 4–5 (n=7) and 6–8 (n=5).
*Conditions:* MSK/pain (n=11), cardiopulmonary (n=8), fatigue/post-viral (n=6), cancer (n=3), neurological (n=2) and gastrointestinal (n=2).

Interview data for both patients and HCPs were coded into four NPT themes (matching the core domains) and additional non-NPT or ‘other’ themes addressing aspects such as the context (ie, demographics and site services), the intervention’s proposed mechanisms of change in the programme theory and suggested changes to the intervention. All themes and subthemes with example quotes are detailed in online supplemental file 4.

### Patient perspectives

#### NPT themes

##### ‘Making sense of the PERFORM intervention’ (coherence)

Prior to taking part in PERFORM, most patients reported that they engaged in regular light physical activity (eg, walking or gardening). Some reported doing regular exercise already, though most stated that they used to be more active before their conditions developed.

I was managing, like, ten … maybe 10, 15 minutes of walk … That’s the most exercise I’ve had. But nothing … nothing like working with weights or treadmills or bikes, you know, nothing like that. (F101)

Very few participants had concerns about the exercise component of PERFORM, and most had no expectations or were happy to be taking part. Patients understood that regular exercise is important for general health and managing their conditions. Barriers to patients managing their conditions before taking part in PERFORM included complex and fluctuating symptoms, stress and other lifestyle factors such as poor sleep, and inadequate access to or support from healthcare professionals, for example, not being taken seriously or being unable to access appointments. Facilitators of managing conditions before PERFORM included medication and management strategies (eg, pacing for fatigue), support from family members and HCPs, and, for a minority, regular exercise and fitness.

Yeah, friends and family are great with it and take it into account and everything, are aware of the conditions … friends know the situation and take care of me if there is an issue like that [low blood sugar]. (F109)

##### ‘Understanding the value of PERFORM’ (cognitive participation)

Most patients were motivated to take part because they wanted to improve their symptoms, and general health and well-being. Other reasons included needing motivation and confidence to exercise, wanting to contribute to research and feeling that they had nothing to lose by trying.

I just thought I would give it a go and what have I got to lose. You can opt out at any point. You have got nothing to lose really. (F103)

All patients believed the programme had value because of the holistic approach and would recommend it to others.

So, it was more on your overall situation with a bit of physical exercise included really sometimes, that was the emphasis, it certainly wasn’t just, sort of, like, 90 per cent physical exercise. (F116)

##### ‘The work of taking part in PERFORM’ (collective action)

Barriers to engaging in PERFORM included difficulty with the timing or frequency of sessions (eg, fitting around work schedules or family commitments) and symptoms flaring. Four participants also experienced transport and parking challenges that resulted in missing sessions, such as relying on others for lifts and a lack of accessible parking at one hospital site.

… it was two days a week, whereas I could only make one day because the other days my wife was at work. I tried to get a friend of mine to get me to the hospital and get me back … but then they had something come up in their family commitments, so they weren’t able to do that for me. And I couldn’t afford a taxi ride from home to the hospital, bus is just too long, so, you know, that didn’ t work out. (F101)

Facilitators of engaging in PERFORM included having a positive attitude and committing to the programme, knowing that it was a safe environment (eg, if anything went wrong with blood pressure) and support from others (eg, a friend attending as support or flexibility from work).

You just plod along and do it. You think to yourself, you have made a commitment you need to fulfil that commitment. (F103)

##### ‘Reflecting on PERFORM and its impact’ (reflexive monitoring)

Participants reported that the supervised exercise sessions were flexible, allowing them to go at their own pace and do what they could manage, while still offering challenges to keep them motivated. All participants felt that the HCPs were attentive, encouraging and supportive and made a difference to their experience.

The facilitators were really good … And they're very friendly. And I think when you come into an environment like that and you’ve got health conditions, you do worry about looking like an idiot, I suppose. And they, kind of, put you at ease and it really does make the session a very nice place to be in. (F102)

Patients appreciated the opportunity to use equipment and learn how to use it safely and effectively in a supervised environment. At one site, session pacing was sometimes felt to be too fast, or HCPs did not always account for age and ability. Participants generally found the home exercises straightforward and completed them. Six participants reported finding it challenging to exercise at home without the structure and motivation of the supervised sessions or without the same equipment. A minority (n=3) did not do the home exercises at all because they did not appeal or they found it difficult to fit them in, or they chose to do their own activities (eg, cycling).

I have to say to you this, doing them at home isn’t the same at all as doing them with the discipline of the people there and the advice they give you about how you’re performing. It’s not the same. I want to say it’s the same, and I’m doing the exercises at home, but I don’t feel the same about them as I did when I was in the session itself. (F218)

Most participants (n=14) found some aspect of the health and well-being sessions useful (eg, relaxation and breathing strategies). Seven participants reported finding some of the health and well-being sessions unengaging or felt most of the material was not relevant or useful (eg, standard diet education was not relevant for someone with a stoma). These sessions may therefore benefit from changes such as greater flexibility in the format, more guidance for staff on tailoring to the group and leaflets specific to conditions (eg, concerning diet). Some patients (n=4) found the education hard to implement because of variable symptoms such as fatigue and brain fog, which made it difficult to reliably be active at home or remember to make positive dietary choices.

It was useful. Some of the points that came out, you know, I could relate to myself. But some of it, it’s difficult to put it in practice, just because of, like, how my body restricts me. (F101)

Just over half of participants (n=11) found the progress tracker useful to monitor changes and create a routine for exercise. For four participants, the tracker did not reflect the improvements they felt, which they found disappointing. Almost half of participants (n=8) either found the tracker difficult and cumbersome to use or did not use it at all, for example, because they did not like writing things down or had dexterity issues.

[The progress tracker] was quite good, yeah. Because when you came to use it, you’d go to the next session and the first thing you do is get that out, don’t you? So you look at what you did before, oh, I’ve got to try and do a bit more than that, and that’ s why you’ re pushing yourself then. That is a great help for pushing you without anybody actually standing there and pushing you. (F113)

I found it disappointing [using the progress tracker], for me, because it was the same from week one to week number six and seven and eight. I would imagine the majority of people would find it a benefit because if they were improving week after week then that would definitely encourage you more. (F103)

All six participants indicated that they found the maintenance sessions useful to discuss progress and catch up socially with the group; however, they felt that there was a lack of clarity on what the maintenance sessions would involve.

Yeah, [the maintenance session] was good. Obviously, it was, you know, what we’ve achieved, what we’ve changed. We spoke about how each other’s feeling, you know, the positive changes, if there’s anything else we could do, what we’d do. So just generally talking about obviously health and wellbeing. And it was nice. It was nice to hear how everyone’s progressed or are progressing that had taken part. It was nice to see some familiar faces that you’re not…you saw while you were doing the sessions as well. (F118)

All participants made some positive behaviour changes around physical activity, exercise, diet and lifestyle. Patients at the community rehabilitation site had also continued at the centre’s gym after completing PERFORM. Participants reported experiencing increased confidence to exercise and manage their health, improved mental well-being, improved symptoms and feeling fitter and more able to be active. Four participants felt little physical impact due to unstable symptoms (eg, long covid), missing sessions or being unable to reproduce the same level of exercise at home.

Doing the pilot study has done exactly what I wanted. It got me into it. I’m carrying on with the exercise and I feel lightyears better than I did in January. Put it like that. I mean, I was at the gym yesterday and I really didn’t want to go, but I made myself go and I ended up doing everything that I normally do. I feel great today. (F311)

Since I’ve been doing PERFORM, they helped me with my breathing, so I don’t panic with it … I learnt how to do the breathing exercises, and they helped a million per cent, really helped. (F313)

I think in terms of improving, I would have liked to get more out of it. Like I said, the more sessions … the sessions I missed, you know, it would have been beneficial to do that and maybe do it twice a week. (F101)

### Other themes

#### Mechanisms of change and maintaining change

Several mechanisms of change present in the programme theory were noted. The social or group aspect of the sessions was overwhelmingly reported as positive and seen as integral to the programme’s success, particularly for participants who experienced isolation because of their health. Groups were reported as friendly and encouraging; patients supported each other and some were keeping in touch beyond the programme.

Although the support was physical, in the gym, it was also mental. You generally felt that you had a community around you. And a caring community. In both the people you were doing it with, other people who were using the gym and the staff themselves. The value of that sense of community is beyond measure really. (F320)

The programme structure and routine helped to build habits, and goal setting helped provide direction and a marker for progress.

What I liked was the discipline of it, I suppose. I liked the fact that it started on time, that you had a set programme that you were given and you worked through your programme with supervision when you needed it. (F218)

I thought that routine was a good thing, and it’s kind of what I’ve carried on since … So Tuesdays, Thursdays or Fridays it tends to be, and Sundays … I think that routine came from the way it worked with PERFORM, so it definitely did get me into a good habit there. (F109)

Most participants (n=13) reported having good support from friends and/or family. One participant was engaged in another study involving tracking day-to-day exercise via an app and reported this helping progress during PERFORM.

Barriers to maintaining change included lack of motivation, time, support and structure, in addition to symptom flare-ups or progressive conditions worsening.

I feel a bit left up in the air now at the end. Yes, I’m continuing with things and I’ve got the weights, as I’ ve already said, but I feel a bit chopped off, if you know what I mean. Where do I go from here, sort of thing? (F218)

Facilitators of maintaining change were focusing on capabilities, taking small steps, maintaining exercise as part of a routine and having continued support and resources (eg, equipment).

It would be nice if there was actually an ongoing programme. 14 weeks obviously covers what the educational side of it is, but the exercise is obviously something you want to keep doing indefinitely, and it would be nice if that was an ongoing thing if there was some sort of session you went to once a week or once a month or whatever and use the kit and the equipment there. (F109)

#### Changes to the intervention and trial methods

Suggested changes to the intervention included making the health and well-being sessions more interactive; making them optional; extending the programme or providing ongoing support; expanding the programme to more people and settings (eg, in the community); making the progress tracker easier to use; providing clearer and more accessible schedule options (eg, later start time); ensuring opportunities to make up missed sessions; more information about the purpose of maintenance sessions; help getting to sessions or reimbursement for travel and ensuring staff are fully aware of patient conditions and circumstances.

I think I’d look very carefully at the sessions at the end, because to keep people’s interest, it’s a long session, an hour, and then another half an hour with the lectures, or whatever you want to call them. I would look carefully at the content of those, and maybe ask some of the participants what they would want to discuss at the end of the session. (F218)

Just 10 o’clock would make all the difference. Because you’re missing the rush hour, you don’t have to get the early bus, you’ve got a good confidence that you’d actually get on the bus and actually get to the session in time. (F116)

Most participants found the trial information sufficient and easy to understand. Assessments were felt to be straightforward; some noted that they were lengthy but generally understood that this was necessary in a trial. Some participants also wanted to see their personal results at the end of the programme to see if they had improved. Suggested changes to trial methods included reiterating information about the research in sessions and reducing the number of questionnaires.

I suppose the checkbox section of it goes on a bit, so it becomes a bit of a drag, levels of satisfaction with different bits of life and stuff. I mean, if there was a way to strip that back a bit, cut back some of those questions it probably wouldn't hurt from my point of view, from the involvement of a participant. (F109)

### Healthcare professional perspectives

#### NPT themes

##### ‘Making sense of PERFORM’ (coherence)

In comparison to usual care, HCPs described PERFORM as giving more equal weight to conditions, being more group based and having more education. However, the exercise component was seen as similar to existing exercise rehabilitation at the sites.

Being involved with PERFORM has been great to enable us to widen the net, so to speak, and enable us to see who we can offer rehab to. Exercise is medicine, we know that, we know it is good for a lot of people and not a lot of people are doing it. So the more people that can the better. (HCPF02)

Most HCPs found the training to deliver PERFORM reasonably thorough and informative and felt prepared. One team felt unprepared for logistical issues (eg, consent process and scheduling), while another HCP found the training too basic and slow-paced for those with advanced experience. Junior staff delivering PERFORM at one site did not attend training and relied on team meetings.

I think they covered all of the key components, really. I suppose I am quite an experienced facilitator and have delivered quite a lot of education to patients in the classes and things that I had delivered previously. I think that although the training was good, there was no assumption of prior knowledge, which for somebody who’s got no experience, that’s absolutely brilliant. (HCPF04)

##### ‘Understanding the value of PERFORM’ (cognitive participation)

HCPs in NHS services described existing rehabilitation as focusing on isolated conditions rather than MLTCs, with patient reach restricted by resource limitations and some patients therefore ‘falling through the net’. Motivations for wanting to use PERFORM included wanting a professional challenge, enhancing patient care and wanting to support research. All wanted to carry on using PERFORM, which had in some cases inspired changes to the existing approach (eg, more focus on providing patients with health education). All HCPs would recommend using PERFORM but advised that staffing and funding capacity must be considered.

I think it would continue to be really positive. The patients had a really good, positive experience, all the staff enjoyed it, myself and [name] both really enjoyed delivering it. And I think if we could keep doing that for patients, I think that would be really important. (HCPF03)

I would say that the experience on the whole has been really positive, but there is a lot to consider. You need to look at the assessment process and if you have got the capacity and the time to be able to deliver those assessments. Then can you fit that and embed it within the service that you deliver? Have you got the capacity to do that? (HCPF02)

##### ‘The work of delivering PERFORM’ (collective action)

Challenges involved in delivering PERFORM included finding staff time, delegating duties and getting co-operation from referring departments. Accommodating the intervention strained other services at one NHS site (eg, having to pause a breathlessness class due to limited staff and space).

We did this at the sacrifice of a class which fits up to 16 people in a week. And again, for a short period of time you can justify it … But I guess denying your service what is quite a big chunk of time, that’ s tricky. And it’s really hard to get people on board then … we still had the same amount of assessments, so we just got rid of a class, but all of our other service stayed the same, so we now just have a bigger wait for class. So, that didn’t make the intervention that we were delivering difficult, but it did make the services that we’re delivering more challenging. Things like this are always going to be at the sacrifice of something else. (HCPF01)

Tailoring to individuals in the groups was also challenging for the community rehabilitation site, as their usual approach was one-to-one. Facilitators of delivering PERFORM included having a supportive and enthusiastic team, being able to fit in sessions easily and strong support from the research team.

Things that helped me to deliver the intervention were the fact that we had a really strong team behind us, so the support for the trial throughout was actually very good, and the little touches like the newsletter to keep us all up to date, that was really helpful, and actually, the celebration when we hit milestones, and keeping everybody up to date with how the other different trial centres were getting on, that was all really, really positive. (HCPF04)

##### ‘Reflecting on PERFORM and its potential’ (reflexive monitoring)

HCPs overall found the health and well-being sessions engaging and useful but felt that some topics were too prescriptive or not relevant to some patients (eg, breathlessness advice for those with no breathlessness). Staff found PERFORM useful to grow their professional skill set and confidence working with MLTCs, and the programme illustrated how to broaden the reach of rehab. The intervention format was adhered to as far as possible, and exercise was adjusted as part of normal tailoring. Health and well-being sessions had useful guides and prompts, but HCPs sometimes needed to adjust or update information to make it relevant to all in the group.

I think they just found sometimes the relevance of it, of some of the teaching stuff quite difficult, so they maybe got almost questioned why they had to listen to something to do with, like, stress busting when they don’t have stress and stuff like that … so we did have sometimes to explain that’s part of the course … it might equip you with the tools so if you’re ever faced with something like that in the future, you have that tool to manage that. (HCPF05)

HCPs found that patients were enthusiastic and supportive of each other and keen to participate. Patients appeared to enjoy the sessions and reported noticeable improvements. The programme appeared to empower patients to make small changes, and the social support element of the intervention was noted as key to its success. Some patients struggled to attend or dropped out, for example, due to health flare-ups or scheduling conflicts.

I think the most value was them, kind of, supporting each other … it did become a really nice supportive environment and everyone, kind of, celebrated everyone’ s success and progress and would ask for each other, where’ s so and so today, is everyone okay, and that was really nice. And when someone was finishing, I remember people applauding. (HCPF01)

Several challenges to sustaining PERFORM were noted. In the NHS, one department taking the load or running the intervention can potentially strain the wider service, and adequate staffing and funding would be a challenge. HCPs at the community rehabilitation site felt that it may be difficult to manage patient expectations in a paid-for service. There were also concerns that the selection criteria may be too broad if the programme is implemented, making it difficult to judge the cut-off point, that is, too many patients eligible who do not necessarily struggle with their conditions, which HCPs found with some patient participants in this study.

To be able to deliver it across the board and get more patients through an exercise and advice and education programme, we do need the resource to be able to do that. And, you know, my services who offer these sorts of classes already are facing waiting lists. (HCPF04)

Facilitators of sustaining PERFORM included supports such as more funding, staff and physical space and having data to show funders the benefits. HCPs believed that maintaining good training and sharing learning from implementation would help rollout, and in the NHS, buy-in from other departments and joined-up referral routes would be necessary. It was also highlighted that the programme would need to ensure GP or specialist support and follow-up would be available for patients.

We need more space. As I say, we sacrificed pulmonary rehab class, that’s because we don’t have anywhere else to put it, we don’t have enough space anyway. So, I think commissioners really need to think about that, how people can go on to deliver these services. (HCPF01)

## Other themes

### Changes to the intervention, training and trial methods

Suggested changes to the intervention included (1) encouraging staff to be more creative with exercises; (2) ensuring education information is up to date and relevant; (3) building in more tailored discussions to health and well-being sessions; making exercise paperwork more flexible and practical (eg, allowing room to make adjustments or write additional information); and (4) providing more detailed guidance on the exercise, how to deal with control participants (ie, what information was acceptable to give them) and how to administer assessments in the training.

There were a few things that we had to iron out after that we then had to have a lot of back and forwards with. So, perhaps we could have been more prepared upfront before going to the training so that we could have ironed them out on the day, rather than what I think we did was all just turned up not really knowing what to expect, had all of this information and then had to digest it later. (HCPF01)

HCPs reported that the trial procedures were straightforward overall, with some challenges. It was difficult to find time for assessments and trial administration and to complete assessments and scheduling to allow groups to start and finish together. Non-clinicians/researchers conducting screening created more work for one NHS site (ie, screeners still needed to check criteria and specific patients with HCPs), and audio recording sessions and assessments were difficult logistically (eg, if participants did not consent). HCPs reported struggling to manage sudden paperwork changes (ie, new forms or versions) and moving paper copies over to digital, which was very time consuming. One HCP also found it challenging as a clinician to deal with control participants’ disappointment and not knowing how much guidance to give them without influencing trial results. Suggested changes to the trial methods included having pretraining materials (eg, clarifying expectations), involving clinicians in screening and reducing the assessment burden.

I suppose the questionnaires because there are so many of them, I suppose to know whether obviously they are all essential or not and whether that could be adapted slightly. Again, that is I suppose part of doing the initial feasibility, isn’t it, to see whether everything is necessary and to review the outcomes of those. It was a lot of information for patients to process during an assessment. (HCPF02)

### Programme theory

The key mechanisms in the initial programme theory were largely supported and are summarised below, with one addition suggested based on our data.

#### Building autonomy and empathy

Most participants felt they had built a degree of autonomy in exercise and self-management and felt more confident, though participants at both NHS sites reported a lack of ongoing support as a barrier to maintaining change. This was not an issue for those at the community rehabilitation site, who were able to continue at the site’s gym with the same staff and meet up with some of the same group members.

#### Tailoring exercise and self-care support

Participants felt that sessions were appropriately tailored to their needs, allowing them to go at their own pace. The only exception was some patients reported the pacing was too fast at one site, where staff reported being less confident tailoring to individuals in the groups.

#### Self-regulation

Patients found that the process of goal setting and reviewing progress provided direction and helped them to build confidence in self-managing, though this aspect was not often raised by patients as a key facilitator or mechanism of the programme.

#### Building a functional ‘illness model’

Patients reported feeling more confident in their ability to manage their conditions using the skills and strategies learnt in the programme. For example, several mentioned the relaxation techniques as helpful for their well-being, even if they had not felt particularly stressed previously.

#### Psychological adaptation to living with chronic conditions

This mechanism was not a key concern for patients. While they reported feeling more confident, most had been living with their conditions for long periods. Two patients with newer diagnoses (long covid and neuropathy) reported still struggling to adjust to living with their conditions and were still experiencing anxiety and feelings of loss. It is possible that health and well-being sessions did not allow for sufficient tailoring to address different levels of acceptance among group members.

#### Engaging/building social support

The social aspect of the sessions was overwhelmingly reported as positive and integral to the programme’s success. Groups were friendly and encouraging, and patients supported each other. Most participants had support from friends and/or family and all highlighted the importance of the encouragement and support from HCPs.

#### Cognitive behavioural strategies

Patients reported improvements in mental well-being and in their ability to manage both day-to-day stress and the stress or anxiety of living with long-term conditions. The breathing technique was frequently mentioned as a straightforward and memorable way to slow down when feeling stressed or anxious.

#### Other factors

A further mechanism mentioned frequently in interviews but not in the original programme theory was the benefit gained from the programme’s structure and routine. Patients found the regular structure of attending the sites in person, and for some using the progress tracker, gave them the motivation and momentum that they struggled with on their own. The structure and routine helped patients to build day-to-day habits around both exercise and lifestyle changes, such as setting aside time for the home exercises and being more proactive about increasing general activity. Some chose to continue exercising on the same programme days after completion to make it easier to maintain the routine. Sticking to a regular routine was also highlighted as a facilitator of maintaining progress.

In terms of co-interventions, one participant started a falls programme shortly before PERFORM completion, while another was engaged in a separate study prior to and during PERFORM that involved tracking day-to-day exercise via an app; the latter reported the additional tracking helping their progress on PERFORM.

## Discussion

### Key findings and implications

Patients and HCPs reported favourable experiences with the PERFORM intervention and felt it was valuable for managing symptoms of MLTCs. Patients reported that they experienced improvements in physical and mental well-being and that they made efforts to enact positive behaviour changes. The programme theory principles and mechanisms were supported, with one additional mechanism reflecting the role of structure and routine in creating habits was highlighted in the analysis. Several barriers and facilitators to engaging in or delivering PERFORM and the trial were identified that should be addressed in a future full RCT and implementation, particularly focused on issues of resources and logistics, for example, staffing levels required to deliver the intervention and the trial assessment burden. There were pronounced differences between the two site types (NHS hospital and private community rehabilitation clinic) in several areas, most notably in available resources for patients to have ongoing support to maintain exercise and social support.

Based on patient and HCP suggestions, several changes have been made to the PERFORM intervention and training in preparation for a full trial. Regarding the training to deliver the intervention, HCPs will be provided with pretraining information (ie, what PERFORM is and the trial methods) to allow them to familiarise themselves with the intervention and the trial expectations before the training itself. This will allow more time to consider specific needs or challenges at their sites and clarify queries at the sessions.

Training will clarify expectations, particularly how much flexibility is required or permitted in the exercise and education, how much guidance should be given to control participants without influencing the trial and how to effectively tailor the intervention, with examples of ‘good practice’. Staff will also be given additional training on how to use behaviour change techniques. As junior staff at one site did not attend training, recorded training videos will be made available, and it will be that training is mandatory for staff delivering PERFORM. This online training will also be helpful when there is a change of staff during the trial.

As not all patients can access suitable mainstream gym facilities or classes near them, sites will be asked to more clearly signpost options for ongoing follow-up support (eg, local gym programmes, websites) after the main programme to try to ensure that patients do not lose momentum or confidence. Sites will also be encouraged to offer alternative session times where possible to accommodate patients with work or caring responsibilities.

To ensure the health and well-being sessions are as relevant and engaging as possible, information provided to HCPs will be revised where possible to reflect the needs of different conditions. HCPs will be given guidance in the training, to facilitate more tailored discussion and stronger use of goal setting and action planning. Teams will be encouraged to consider the profile of each group and provide condition-specific leaflets to patients who have more complex needs (eg,history of inflammatory bowel disease). This will allow the content to be more tailored while maintaining the core functions of the sessions.^30 31^ To better support patients to maintain exercise and lifestyle changes, staff will be asked to focus more on behaviour change in the health and well-being sessions.

To further support participants to engage in behaviour change, they will be provided with an online resource of intervention documents (eg, education session materials and exercises) to use after their sessions. As almost half of patients had difficulty with the progress tracker, did not like it (eg, did not show the progress they felt), or did not use it, the progress tracker will be simplified. More subjective questions (eg, perceived difficulty of a specific exercise) will be added to the tracker to ensure that patients see some of their felt progress reflected and HCPs will be asked to encourage use of the tracker and review progress. Participants will also be provided with clearer information about the maintenance sessions, what they will involve and reminders.

Changes that patients and HCPs suggested to the trial methods focused on logistical issues, such as the time commitment and paperwork. Time and resources permitting, HCPs will be asked to offer patients their assessment results at follow-up, to allow them to see any changes. Information about the study (ie, purpose and structure) will be reiterated to patients in the invitation letter, which may also improve engagement and/or retention if participants have a clear understanding of the purpose and potential benefits. As some patients missed sessions and wanted to make these up, HCPs will be reminded in the training that this should be arranged where possible.

While patients and HCPs understood the need for numerous assessments in a trial, the time burden was still felt to be high, particularly for staff. We intend to reduce the number of outcome measures in the full trial, and patients will be allowed to complete core questionnaires at home in advance of the visit. As a small number of participants reported not struggling with their conditions or general health and fitness prior to PERFORM, we have adjusted the patient eligibility criteria to ensure a ceiling of fitness, for example, breathlessness symptoms when hurrying on level ground or walking up a slight hill. We will perform a subgroup analysis of outcomes in the full trial to assess whether the eligibility criteria are specific enough to be sustainable in any future rollout. Due to potential differences between site types (NHS and community), we plan to complete an exploratory subgroup analysis in the full trial exploring the difference between these two contexts. Healthcare professionals will also be asked to complete three questionnaires that will provide more detailed information about their professional experience, existing site services and views on exercise rehabilitation. This will consist of a clinician survey completed at baseline and follow-up and a site rehabilitation service checklist developed by the team leading the planned PERFORM Australia trial.

### Considerations for implementation

Potential barriers and facilitators of implementing PERFORM are consistent with those identified in the wider implementation science literature,^32^,^35^ with a particular focus on resources and logistics. Buy-in to the intervention was not a barrier for the HCPs we interviewed; they were enthusiastic about PERFORM and saw the benefits for patients. However, the NHS faces significant logistical barriers to implementing new interventions that cannot be fully resolved at the level of a single programme.^36^ Sites may struggle to find the time, staff with relevant experience and space to deliver PERFORM, due to widespread pressures and limited resources. Although this was more straightforward for the feasibility study as it was only temporary, one NHS site still needed to pause a breathlessness class to accommodate PERFORM due to limited space and staff. Such issues will need attention in any future implementation both in a fully powered trial or roll out following a positive result in such a trial, and external resources or partnerships (eg, with leisure centres) may need to be utilised to access the space and facilities lacking in the NHS. These challenges will be investigated further in the full trial by interviewing managerial staff.

There may also be challenges related to health services often operating in siloes, whereby patients are referred to single-condition services.^37^ Referring departments may be reluctant to refer patients to PERFORM or may not have capacity to consider alternative rehabilitation approaches. Additional work may therefore be required to prime public health services for PERFORM and shift the culture towards a more holistic way of working with people with MLTCs.

The community rehabilitation site did not experience the same logistical challenges of staff, scheduling and space, as they are a dedicated exercise rehabilitation centre and already take an MTLCs approach. This type of context also allows for ‘wrap-around’ ongoing support, as participants were able to continue using the gym with supervision from the same staff who had supported them during PERFORM. The centre also has a dedicated space to allow patients to socialise with each other and the staff, maintaining the social connection and social support that is a core mechanism of PERFORM. However, there could be challenges integrating private community site data with NHS data. These are important factors to consider as they may impact the long-term effectiveness of PERFORM for those attending different types of sites, and they also have implications for the future implementation and sustainability of PERFORM.

### Strengths and limitations

This study evaluated an innovative exercise programme for people with MLTCs, highlighting key areas for improvement for a full RCT and potential barriers and facilitators of implementing PERFORM. The use of NPT and the programme theory to guide the interviews and analysis and provided a strong framework to uncover the work required to take part in or deliver the intervention and the analyses identified required changes to the programme theory. Interview data were also double coded, supporting robust analysis.

There are several key limitations of this study. Although we made every effort to sample purposively and achieved a reasonably balanced spread of age, gender, conditions and other demographic characteristics, this was limited by the number of participants who consented to be contacted and who were then available for interview. The participants who agreed to be interviewed may have had a more positive experience of the intervention than those who did not consent. Despite efforts to do so, we were also unable to interview participants who had withdrawn from the study (eg, due to illness or personal circumstances), who may have provided different views or experienced less improvement.

Patient participants were skewed towards those who were older and retired, potentially due to both the higher incidence of MLTCs in this group and the logistical challenges for people with work and caring responsibilities. HCP interview data were limited by the relatively small number of sites and HCPs who were actively involved in intervention delivery. While HCPs provided valuable insights into the potential barriers and facilitators of implementing PERFORM in future, we did not interview leadership teams at any of the sites, which may have produced a more detailed understanding of the challenges facing sites.

Response bias is a risk in any qualitative research, and this study is no exception, as participants may have wanted to provide socially desirable or positive responses. Attempts were made to mitigate this by the interviewer having no involvement in intervention recruitment or delivery and encouraging participants to be honest. Participants were assured that their responses would be anonymised in both the informed consent process and at the start of the interview.

## Conclusions

The results of this study support our plan to undertake a UK multicentre RCT to assess the clinical and cost-effectiveness of the PERFORM intervention. The intervention and study design were acceptable and feasible for the patient participants and HCPs interviewed. All found PERFORM to be a useful intervention and reported positive impacts. The group or social aspect of the programme, structure, education and support from the HCPs were the positive mechanisms most mentioned by patients as helping them to complete the programme and improve self-management. All patients and HCP participants reported that they wanted to continue using the PERFORM intervention and would recommend it to others. Key barriers or challenges for HCPs at NHS sites were organisational issues including scheduling, staffing and space limitations, which require consideration for a full trial and future implementation into routine practice.

## Supplementary material

10.1136/bmjopen-2025-100199 online supplemental file 1

10.1136/bmjopen-2025-100199 online supplemental file 2

10.1136/bmjopen-2025-100199 online supplemental file 3

10.1136/bmjopen-2025-100199 online supplemental file 4

## Data Availability

Data are available upon reasonable request.
