# Supplementary material for: Personalised Exercise Rehabilitation FOR people with Multiple long-term conditions (PERFORM): findings from a process evaluation of a randomised feasibility study
Source: BMJ Open. 2025 Sep 17;15(9):e100199. doi: 10.1136/bmjopen-2025-100199 (PMC12458778; doi:10.1136/bmjopen-2025-100199)
Supplement: online supplemental file 1 [file bmjopen-15-9-s001.docx]

**Appendix 1: PERFORM logic model**

**Context**

- Participant characteristics (e.g., age, gender, ethnicity /cultural background,) and personal circumstances (e.g., types of long-term condition (LTC), rural/urban location, access to transport, disposable income, education, prior experience of living with LTCs).
- Site characteristics (e.g., availability of resources, appetite for change, buy-in from staff)

**Intervention**

PERFORM is an *‘exercise plus self-care support’* programme to improve physical and mental wellbeing in people with multiple long-term conditions. It includes:

1. A group exercise class where attendees learn how to exercise safely and effectively
2. A self-care education programme to help them manage common symptoms and promote ongoing exercise and physical activity
3. Support to maintain self-care behaviours in the long term.

**Intended processes of change**

- Empathy building /engagement
- Building understanding /illness model /outcome expectancies /personal role in self-care
- Behavioural self-regulation (planning, review of progress, review of plans)
- Emotional self-regulation (managing acute stress /anxiety /low mood, cognitive-behavioural regulation)
- Building social support /social approval for self-care
- Building competence /confidence to enact both change techniques and self-care behaviours.

**Long term outcomes**

- Sustained/ongoing physical activity and self-care behaviours
- Improved quality of life
- Reduced hospital admissions and other healthcare costs

Maintenance of PA and self-care change at 12 months will be moderated by perceived autonomy, competence, relatedness, the perceived benefits of changes in self-care (social, physical, emotional) and (for PA only) enjoyment of PA.

Change in health-related QoL outcomes will be mediated by changes in PA and other self-care behaviours.

**Short term outcomes**

*Self-care behaviours*

- Increase in physical activity
- Using taught self-care strategies to manage symptoms (e.g., mindfulness techniques to manage stress)
- Engaging in social and pleasurable activities

*Determinants of self-care*

- Processes of change (listed opposite)

*Physical /mental wellbeing*

- Reduced burden of common symptoms (e.g., pain, fatigue, low mood, perceived stress, breathlessness)
- Improved sleep quality

*Increased quality of social support*

**Intervention delivery**

Adequate fidelity of delivery*, including:

- Facilitators use a person-centred communication style
- Personalised tailoring of exercise prescription
- Planning for long-term physical activity and self-care
- Review of progress and problem-solving
- Engaging /enhancing social support).

Engagement /attendance

**Feedback loops**

Participant attendance, use of BCTs, changes in self-care (inc. PA) are reinforced by perceptions of benefit (emotional, physical and social), as well as changes in perceived autonomy, competence and relatedness and (for PA only) enjoyment of PA. These interactions can build into positive cycles of perceived benefit and behaviour change, but may be mediated by delivery quality and intervention attendance.
