# Supplementary material for: Personalised Exercise Rehabilitation FOR people with Multiple long-term conditions (PERFORM): findings from a process evaluation of a randomised feasibility study
Source: BMJ Open. 2025 Sep 17;15(9):e100199. doi: 10.1136/bmjopen-2025-100199 (PMC12458778; doi:10.1136/bmjopen-2025-100199)
Supplement: online supplemental file 2 [file bmjopen-15-9-s002.docx]

**Appendix 2: PERFORM patient interview schedules**

**Context**

*Participants’ circumstances, understanding of their conditions, and how they managed their conditions before taking part in the programme.*

- Can you tell me a bit about your living situation?
  - Do you live in a city or rural area?
  - Do you live with anyone at home?
  - How did you travel to the programme sessions? Do you have access to a car or public transport?
- Can you briefly tell me about your health conditions and the symptoms you experience?
- Before taking part in the study, what helped you to manage your conditions? e.g. friends/family, getting appointments/advice from HCPs, employment, benefits/carers, home adaptations, prescriptions
- What got in the way of being able to manage your conditions?
- Had you taken part in any other exercise or lifestyle interventions or support programmes for any of your conditions before this study?
  - What did these involve?
  - What did you think of them? (Were they helpful/not helpful?)
- Before the study, what kinds of physical activity or exercise were you doing? What were your main reasons for doing this activity (or not doing much)? Did you enjoy doing it?

**Motivation**

*Participants’ motivation for taking part in cardiac rehabilitation.*

- What made you want to take part in the PERFORM study?
  - Why this programme?
- What were you hoping to get out of it?
- What were your initial thoughts about taking part in the PERFORM programme?
  - Any concerns about the exercise/other elements?
  - Any positive features that attracted you to the programme?
- What are your thoughts about the importance of physical exercise for general health?

**Experience of doing the intervention**

*Participants’ engagement with the programme.*

- How did you feel about taking part in the group exercise sessions? e.g. the idea of being in a group setting, time commitment
- How did you feel about the home exercise activities? e.g. finding time and space
- How did you feel about the health and wellbeing sessions?
  - What did you learn?
  - How did they make you feel about making changes in your day-to-day life? e.g. were you inspired/put off?
- How well do you feel you were able to stick to the programme? (i.e. attending all the sessions, doing the home exercises, making other lifestyle changes)

*What participants liked about the programme/found useful/valued.*

- What, if anything, about the programme (exercise and health and wellbeing sessions) did you like or find particularly useful?
  - Please tell me a bit more, and why (e.g. tailored/progressive exercise, social support, self-monitoring, action planning, health professional’s facilitation style/communication, pace, session environment)
- Did the programme make any difference to you or the way you look after your health?
  - In what ways did it affect how you manage your conditions? e.g. doing regular exercise at home, lifestyle changes, managing the stress of living with long-term conditions
  - Did it improve your physical or mental wellbeing in any ways?
  - Has it had any effect on your confidence to manage your health?
- Was there anything about the programme you didn’t like or found difficult? (describe) If yes, why?
- *(Only if at least one maintenance session attended)* What was your experience of the two follow-up maintenance sessions that happened after 3 months and 6 months?
  - If you attended them, how did you find them/were they useful?
  - Were the two sessions different? Was one more helpful than the other?
  - What about them was useful and why?
  - Do you think it made a difference it being in the group again as opposed to one on one?
  - Is there anything you didn’t like or that we could improve or you would like to see added to those sessions?
  - If you didn’t attend them, why not?
- What were the things that helped you take part in the programme activities? e.g. support of family/friends, transport, group setting, other support services such as online/social media groups
  - How did they help?
- What were the things that made it difficult for you to take part in the programme activities? e.g. lack of support, unpredictable symptom flares, level of commitment needed, post-exercise fatigue, pain, transport
  - How did they make it difficult?

*Potential mediators of the effect of the intervention.*

- How did you find working with the health professionals? Were they helpful? Did that make a difference to your experience?
- How, if at all, did the social aspect of the programme affect you? (e.g. being in a group and spending time other people with long-term conditions) Did you feel supported in the group environment?
- Were friends or family supportive or negative about your participation in the programme? (describe)

*Anything else participants did to achieve increased exercise e.g. using an app etc.*

- What things did you do to actively help yourself increase your exercise? e.g. increasing exercise using an app, using reminders on phone or around home to remember exercises or positive lifestyle changes
- If so, how do you think it helped?

*Barriers and facilitators to longer term maintenance of the new behaviours.*

- What do you think might make it difficult to keep going with any changes you’ve made? i.e. keeping them going in the long term. Why?
- What do you think would help you to keep up changes or continue improving?
- How did you find the progress tracker? Was that helpful?

**Future use**

*What participants think could be improved about the programme.*

- What, if anything, would you change about the programme?

**Contamination/co-interventions**

*Whether participants engaged in other programmes/new treatments.*

- Did you take part in any other programmes or treatments while you were doing this programme? If so, what did that involve?
- (If taken part in other programmes/treatments) Do you think it might have affected your engagement with the PERFORM programme? If so, how?

**Experience of the trial procedures**

*Participants’ views of the recruitment process.*

- During the recruitment process, when you were asked if you wanted to take part in PERFORM, was enough information provided/was it easy to understand?
- Were there any aspects of the recruitment and consent process that you found difficult or disliked? If yes, what were they?
- Is there anything that could have improved the process?

*Participants’ views of the research process, i.e. questionnaires.*

- How did you find doing the tests and questionnaires before you started and after you finished the main programme? e.g. time/energy commitment, easy/hard to understand, purpose explained
- Were there any aspects of the tests and questionnaires that you found difficult or that you particularly disliked? If yes, what were they?
- Is there anything you would like to see done differently in a future large-scale trial?
- Would you recommend participating in the full-scale trial to others?

Anything else to add?
