# Supplementary material for: Personalised Exercise Rehabilitation FOR people with Multiple long-term conditions (PERFORM): findings from a process evaluation of a randomised feasibility study
Source: BMJ Open. 2025 Sep 17;15(9):e100199. doi: 10.1136/bmjopen-2025-100199 (PMC12458778; doi:10.1136/bmjopen-2025-100199)
Supplement: online supplemental file 3 [file bmjopen-15-9-s003.docx]

**Appendix 3: PERFORM healthcare professional interview schedules**

**Context**

*Current context and services available at the site.*

- Outside this study, could you tell me your job role?
- What has your role been in this study?
- What is your experience of using exercise in rehabilitation?
- What rehabilitation services are normally available at your site?
  - Who is involved?
- How well do you think your pre-existing (not PERFORM) rehabilitation services work for people with multiple long-term conditions?

**Acceptability and feasibility**

*Acceptability of the intervention elements.*

- In what ways, if any, is the PERFORM intervention different to what your site has offered before to manage multiple conditions?
  - How?
- What was your experience of the training to deliver the intervention?
  - How prepared did you feel to deliver the intervention following the training?
  - What else, if anything, do think it should have covered?
- Can you tell me what motivated you to want to work with the intervention?
  - Tell me more about why / what fed into this high/low level of motivation.
- What, if anything, about the intervention programme itself did you like? e.g. tailored/progressive exercise, strategies, lifestyle support, self-monitoring, action planning
- What was particularly useful?
- What about the intervention, if anything, did you not like or find difficult to deliver?
- In what ways, if at all, did your views or feelings about the intervention change over time?

*Feasibility of delivering the intervention within their context.*

- Can you tell me about how the intervention fitted in with the usual practice of the site/service? (e.g. team setup/staffing, available resources)
  - Could you tell me more about the positive aspects?
  - Could you tell me more about the negative aspects?
- What changes did you need to make as a health professional and as a wider service to be able to accommodate the intervention?

*Barriers and facilitators to implementing the intervention.*

- What were the things that helped you to deliver the intervention? e.g. support from the trial team or colleagues, training
- What were the things that made it difficult for you to deliver the intervention? e.g. lack of support, time commitment
- Assuming that the trial finds the intervention to be effective and cost-effective, what would be the main challenges to sustainability of this service at your site beyond the trial duration?
- What do you think would be the main challenges to delivering this type of intervention more widely across the NHS?
- What do you think would support wider deployment of this type of service across the NHS? e.g. what support would need to be in place, or what changes would need to happen to achieve this?

**Fidelity**

*Intervention fidelity and any changes in delivery of the programme (i.e. not following the manual).*

- How did you find following the format of the exercise component and using the materials? i.e. individual assessment, group exercise sessions, progress tracker, home-based exercise prescription, discharge appointment, 2 maintenance sessions
- How did you find following the format of the health and wellbeing sessions? e.g. Eating for Health, Better Sleep, Managing Pain, Staying Active for Life, Lifting Your Spirits, Boost Your Energy, Stress Busting, Coping Well, Making the Most of Your Medications, Stronger Together, Let’s Relax, Exercise is a Medicine, Breathe Easy
- What, if any, were the changes you made to how you delivered the intervention? i.e. times when you did things differently to what was suggested in the training? (e.g. making adjustments for the needs of individual patients)
  - Tell me more about these changes.

**Future use**

*Suggested improvements to the intervention.*

- What, if anything, would you change about the intervention? (e.g. length, format of the sessions, content of lifestyle sessions)
- What are your views about your service continuing to use the intervention (if possible) with patients with multiple long-term conditions?
- What would you say to a colleague who wants to use PERFORM in their service?
  - What advice would you give them?

**Impact**

*Reflections on the potential impact on patients.*

- How did patients react to the intervention?
  - In what ways did your patients find it helpful?
  - In what ways did patients find it problematic?
- In what ways, if at all, do you think the intervention helped participants to manage their conditions?
  - e.g. What behaviours do you think patients changed?
  - How permanent do you think these changes are likely to be?

*Contextual aspects that may influence intervention delivery or impact.*

- What, if anything, do you think might have influenced the effects of the intervention on patients? e.g. social support, nature/severity of the conditions, other resources
- How do you think those factors might have had an impact?

**Trial procedures**

*Acceptability of trial procedures.*

- How did you find the trial procedures?
  - time commitment,
  - completing trial-related tasks, e.g. doing assessments, doing the training, screening patients
- Not including the intervention related tasks, can you tell me about any aspects of being involved in the trial that you found difficult?
  - Or any procedures that you particularly disliked?
- What were the parts of the trial that were easy to do?
- If you were involved in a future large-scale trial, is there anything you would like to see done differently related to the trial methods?
