# Supplementary material for: Personalised Exercise Rehabilitation FOR people with Multiple long-term conditions (PERFORM): findings from a process evaluation of a randomised feasibility study
Source: BMJ Open. 2025 Sep 17;15(9):e100199. doi: 10.1136/bmjopen-2025-100199 (PMC12458778; doi:10.1136/bmjopen-2025-100199)
Supplement: online supplemental file 4 [file bmjopen-15-9-s004.docx]

**Appendix 4: PERFORM process evaluation qualitative results**

**Table 1: Patient themes summary**

| **Theme** | **Sub-theme** | **Example quotes** |
| --- | --- | --- |
| Making sense of PERFORM (coherence) | Expectations and preconceptions about PERFORM | "I was hopeful in the sense that there would be people there around that you could always ask questions to if needed." |
|  | Experience of other programmes or interventions | "I went on an exercise programme at the gym which was supposed to be supervised. Somebody was supposed to tell you what to do the next time and everything else. It never happened, it never worked. Because it was a local council gym, nothing to do with the NHS, the staff just weren’t there. You just did what you thought yourself, which wasn't ideal.” |
|  | Barriers to managing health pre-PERFORM | “If I went to the doctors or anything, they just say, phone the number 111 or whatever it is … keep a check on it and that’s it. You know, or get yourself to Accident & Emergency, you know, I can’t walk … And I understand that, I understand ambulances are busy, but that’s the part that frightens me." |
|  | Facilitators of managing health pre-PERFORM | "Yeah, friends and family are great with it and take it into account and everything, are aware of the conditions … so friends know the situation and take care of me if there is an issue like that." |
|  | Views on exercise and past experience | “I think it’s really important to have the right balance, at least a little bit, you know, everyone should do some kind of exercise because I think it’s really important to stay physically active for your own health.” |
| Understanding the value of PERFORM (cognitive participation) | Reasons for taking part | "I wanted to participate and see, you know, if there are any real benefits that come out of this and that can improve my symptoms and my health overall." |
|  | Value of the programme and trial | “I think it’s a good programme and I think it definitely has a lot of benefits that could be…if done properly, rolled out to definitely a wider group of people. Because obviously there are quite a lot of people around in this country who do have lots of health issues.” |
| The work of taking part in PERFORM (collective action) | Barriers to engaging with the programme | "It was awful because I was…I'm juggling a full-time job and, kind of, having to make up the extra hours, ‘cause I’ve got to include travelling time as well to and from the hospital. Having to make up the extra hours while you're constantly exhausted was really difficult. And then exercising while you're exhausted as well was…so I did struggle with it.” |
|  | Facilitators of engaging with the programme | "...the support that you had there made…the support having had…they made me feel safe, so I knew that if my blood pressure was going to go through the roof then I was in the right…you know, had people around me to look after me. I knew that if I suddenly had an asthma attack or one of my bones decided to break, fine, ‘cause there’s going to be people there." |
| Reflecting on PERFORM and its impact (reflexive monitoring) | Changing behaviour | "I’ve started aiming for the 6,000 steps a day from getting a cheap treadmill, which I got because I liked using the treadmill on the PERFORM programme. So it’s entirely consequential. I wouldn't be doing that if I hadn’t done the programme. As things stand now, I’m feeling that I can keep with that and probably actually up the steps over time." |
|  | Physical and mental health impact | “But since I’ve been doing PERFORM, they helped me with my breathing, so I don’t panic with it. Especially then, now it’s a lot better, but at that time I used to panic, and when you panic, then obviously you’re gasping for more air. So, I learnt how to do the breathing exercises, and they helped a million per cent, really helped.” |
|  | Programme environment and support | “The facilitators were really good. They were really good. And they're very friendly … And they, kind of, put you at ease and it really does make the session a very nice place to be in.” |
|  | Views on health and wellbeing sessions | "A lot of the stuff I knew already. They were quite well done but a lot of the ground… You see, they would talk about food, for example, and when you’ve got a stoma, your diet has to completely change. So a lot of it wasn’t relevant to me but that wasn’t the session’s fault, I was just somebody with a different kind of system who needed different information, if you know what I mean." |
|  | Views on the exercise component | "I have to say to you this, doing them at home isn’t the same at all as doing them with the discipline of the people there and the advice they give you about how you’re performing. It’s not the same. I want to say it’s the same, and I’m doing the exercises at home, but I don’t feel the same about them as I did when I was in the session itself." |
|  | Views on maintenance sessions | "Yeah, it was good. Obviously, it was, you know, what we’ve achieved, what we’ve changed. We spoke about how each other’s feeling, you know, the positive changes, if there’s anything else we could do, what we’d do. So just generally talking about obviously health and wellbeing. And it was nice. It was nice to hear how everyone’s progressed or are progressing that had taken part.” |
|  | Views on the progress tracker | "It had what you did, for example a bike, how long you were on it, what speed you did and how it made you feel, whether it was hard work … Even though in myself I was breathing better, but the effort to do it and speed I could do and things all stayed the same.” |
| Mechanisms of change | Social aspect of the intervention | “Although the support was physical, in the gym, it was also mental. You generally felt that you had a community around you. And a caring community. In both the people you were doing it with, other people who were using the gym and the staff themselves. The value of that sense of community is beyond measure really.” |
|  | Structure, routine, and other interventions | “I thought that routine was a good thing, and it’s kind of what I’ve carried on since. I’ll be doing the weights this afternoon after this, and I’m working on the same basis again. So Tuesdays, Thursdays or Fridays it tends to be, and Sundays, three days again. I think that routine came from the way it worked with PERFORM, so it definitely did get me into a good habit there." |
|  | Support from others | “My family, immediate family think that I do very well and they encourage me, they’re very supportive of me in having a go, just have a go.” |
| Maintaining change | Barriers to maintaining change | "Well, the main thing is the flare-ups. When the flare-ups kick in, you know. As I said, like, you know, there were some useful points that came out, but to try and put that into practice … especially when the flare-ups kick in … It’s not in my control to stop that from happening." |
|  | Facilitators of maintaining change | "I suppose if there was some sort of ongoing contact, I don’t know, every three months or whatever, then I think maybe that would motivate me, so that by the next contact you’re still on the ball and still going." |
| Changes to the intervention | Access, resources, and support | "What would I change about the progress checker? Perhaps I would make it more detailed. Say, for example, instead of having just bike for ten minutes level one and it hurts, you can put rate on a scale of one to ten how much it hurts and see if it did the same the next day and the next time." |
|  | Structure and schedule | "Even if it was just once a week somewhere [as an ongoing programme], it keeps you doing it. If you are doing it once a week you would do it more at home, I think." |
| Trial methods | Understanding the trial processes | "Yeah, it was all straightforward. I mean, obviously I learned a lot more when I went on the pre-trial assessment and so on. But yeah, I knew plenty up front before that. I knew everything I needed to decide to do it and what I needed to do on the day." |
|  | Trial assessments and questionnaires | “I think I had to ask him, the guy who did it, at the assessment. I think I said, well, where are the stats from the beginning so we can compare? And then he had to look on the machine to find them.” |
|  | Changes to trial methods | "I suppose the checkbox section of it goes on a bit, so it becomes a bit of a drag, levels of satisfaction with different bits of life and stuff. I mean, if there was a way to strip that back a bit, cut back some of those questions it probably wouldn't hurt from my point of view, from the involvement of a participant.” |

**Table 2: Healthcare professional themes summary**

| **Theme** | **Sub-theme** | **Example quotes** |
| --- | --- | --- |
| Making sense of PERFORM (coherence) | How PERFORM relates to existing care | "I think it just gives equal weighting to the different conditions, where we definitely don’t do that in, kind of, our pulmonary rehab services." |
|  | Past exercise rehabilitation experience | "I did work in a musculoskeletal setting, and part of my rotations would have covered MSK, neuro and respiratory, and all of those would have incorporated exercise in some way." |
|  | Training to deliver PERFORM | "Yeah, the training itself was really good and very thorough." |
| Understanding the value of PERFORM (cognitive participation) | Motivation for taking part in PERFORM | "Yeah, I guess I’m very enthusiastic about research. I think I was just on board with the idea. It made complete sense when it was explained, like, of course why wouldn’t we do something like that. So, it just felt like a bit like a no-brainer, so then it just felt easy to be a part of." |
|  | How existing service works for MLTCs | "I guess because the way our services are structured we are really looking at one disease, or certainly if they’ve got joint respiratory diseases or cardiac and respiratory disease we were looking at multiple. But on the whole if they had, like, a musculoskeletal condition we would be aware of it but we wouldn’t consider ourselves as treating that.” |
|  | View on continuing PERFORM | "It works well for us because it is very similar to what we do anyway, so that wouldn’t be an issue.” |
|  | Advice for using PERFORM | "I would say that the experience on the whole has been really positive, but there is a lot to consider. You need to look at the assessment process and if you have got the capacity and the time to be able to deliver those assessments. Then can you fit that and embed it within the service that you deliver?” |
| The work of delivering PERFORM (collective action) | Challenges of delivering PERFORM | "I think the hardest thing is losing your own service space. We did this at the sacrifice of a class which fits up to 16 people in a week. And again, for a short period of time you can justify it, like we’ll get this done within however many months and we’ll be back doing our own thing. But I guess denying your service what is quite a big chunk of time, that’s tricky. And it’s really hard to get people on board then." |
|  | Facilitators of delivering PERFORM | “I think the PERFORM team were always ready to answer our questions … If we had any queries, they were always answered very quickly, which was really reassuring for us as a team delivering it. We didn’t feel on our own delivering it, there was always somebody there to offer a response or come up with a solution.” |
| Reflecting on PERFORM and its potential (reflexive monitoring) | Views on the intervention | "We don’t have to really fix on specific conditions. You want to get overall good health messages out there. You don’t need to tie down to specific conditions. That has been really useful from a clinical viewpoint because it is not until you actually start delivering it that you realise how that works for the patients because you get their feedback." |
|  | Following the intervention format | “The only times that I did go against materials is like I said about a couple of these things on the education. I think one of them was suggesting low fat alternatives and I’d just given a five-minute talk on why low-fat alternatives aren’t as good as they think they are. Just really minor things like that that I would have said differently. They would have been the only times I was not delivering to the, kind of, standard.” |
|  | Patients' responses to PERFORM | "Some people were coming and they have got all sorts of different conditions and actually saying, I have noticed a difference, I am now doing this or I can do this, I couldn’t do that before, it has changed how I feel and think about things, mentally I am in a different space." |
|  | Mediating factors for patients | "They all refer to it as the exercise, but I think the most value was them, kind of, supporting each other, which they perhaps didn’t notice that they were even doing a lot of the time." |
|  | Challenges to sustaining PERFORM | "I think there would be the shared concern around staffing it, funding it, who’s going to pay for that, and what the implications would be on other services. I think they would be the main challenges." |
|  | Facilitators of sustaining PERFORM | "I guess just demonstrating the long-term benefits, what the implications of this will be. Because if it means we’re just going to see these people every year for the next 20 years that’s not probably what we’re trying to do and that’s not easing any burdens anywhere else actually. It’s I guess selling the trade-off, how this benefits the wider NHS would be helpful." |
| Trial methods | Trial procedure management | "I think fitting in the assessments was challenging. So, we led those as a clinical service, but we didn’t screen them, they were screened in research; and I’m not sure that worked in the best way really. And I think probably because we were doing them in addition, so we were really just squeezing them in, so we really didn’t have the capacity to do that." |
|  | Changes to trial methods | "I suppose the questionnaires because there are so many of them, I suppose to know whether obviously they are all essential or not and whether that could be adapted slightly … It was a lot of information for patients to process during an assessment." |
| Changes to the intervention and training | Exercise and health and wellbeing sessions | "I think building on those opportunities to involve the group and make those discussions meaningful to each of those patients was the thing to, kind of, keep building on, and actually, absolutely having the opportunity to be able to do that." |
|  | Scheduling and paperwork | "I think the paperwork perhaps didn’t work as well as it could have, the ones that the patients filled in. It was quite rigid in that, I can’t remember exactly what it said in it, but there was a bit for walking, there was a bit for cycling. Not everyone was able to do these things so we’d do something else … and there just wasn’t space to modify so there was a lot of scribbling out.” |
|  | Training changes | "Perhaps we could have been more prepared upfront before going to the training so that we could have ironed them out on the day, rather than what I think we did was all just turned up not really knowing what to expect, had all of this information and then had to digest it later. So, perhaps some pre-training, kind of, materials and time to think about that and digest it before we got to the session where the people that could answer those questions were there.” |
