# Supplementary material for: Personalised Exercise Rehabilitation FOR people with Multiple long-term conditions (PERFORM): findings from a process evaluation of a randomised feasibility study
Source: BMJ Open. 2025 Sep 17;15(9):e100199. doi: 10.1136/bmjopen-2025-100199 (PMC12458778; doi:10.1136/bmjopen-2025-100199)
Supplement: Supplementary file 5 [file bmjopen-2025-100199.reviewer_comments.pdf]

# PEER REVIEW HISTORY

BMJ Open publishes all reviews undertaken for accepted manuscripts. Reviewers are asked to complete a checklist review form and are provided with free text boxes to elaborate on their assessment. These free text comments are reproduced below.

## ARTICLE DETAILS

### Title (Provisional)

Personalised Exercise Rehabilitation FOR people with Multiple long-term conditions (PERFORM): findings from a process evaluation of a randomised feasibility study

### Authors

Brown, Sophie Eleanor; Simpson, Sharon Anne Anne; Greaves, Colin; Daw, Paulina; Dean, Sarah Gerard; Evans , Rachael A; Withers, Tom M; Ahmed, Zahira; Barber, Shaun; Barwell, Gwen; Doherty, Patrick Joseph; Gardiner, Nikki; Ibbotson, Tracy; Jani, Bhautesh; Jolly, Kate; Mair, Frances; Manifield, James R; McIntosh, Emma; Miller, Daniel; Ormandy, Paula; Smith, Susan; Vogiatzis, Ioannis; Waheed, Ghazala; Taylor, Rod; Singh, Sally J; research team, PERFORM

---

## VERSION 1 - REVIEW

---

**Reviewer** 1
**Name** Saco-Ledo, Gonzalo
**Affiliation** Universidad Europea de Madrid Campus de Villaviciosa de Odón, INVESTIGACION Y DOCTORADO
**Date** 18-May-2025
**COI** None

---

An interesting study entitled "Personalised Exercise Rehabilitation FOR people with Multiple long-term conditions (PERFORM): Findings of a process evaluation of a randomised feasibility study" is being considered for publication in BMJ Open. This type of study is of interest to the journal’s readership. Although it is a qualitative study—and such studies are sometimes undervalued—it deserves attention given the relevance of the research area, and this work may provide valuable insights. However, I have some comments that may help the authors improve the manuscript:

1. It is not necessary to include the abbreviation "PERFORM" in the title.

2. The second page of the Background section could be shortened. Consider integrating some of the references into a slightly expanded first paragraph of the second Background section.

3. The information presented in Table 1 could be integrated into the main text; the table itself is not essential.

4. If possible, the results should also be presented disaggregated by patient sex.

5. The manuscript contains too many subheadings, particularly in the Discussion section; these could be reduced for improved clarity and flow.

6. The Discussion and Conclusion sections could be more concise.

7. The reference numbering throughout the manuscript needs to be reviewed and corrected. Please do not include references that have not yet been published.

8. Please include the trial registration number.

---

| | |
|---|---|
| **Reviewer** | **2** |
| **Name** | **Pathania, Monika** |
| **Affiliation** | **AIIMS Rishikesh, Internal Medicine** |
| **Date** | **07-Jul-2025** |
| **COI** | **None** |

---

its overwhelming for the reader with too many aims , too many interpretations. Though the idea is very good but this needs clearly defined methodology . The author should add clarity by addressing primary objective first.

---

## VERSION 1 - AUTHOR RESPONSE

| Reviewer 1 | | |
|---|---|---|
| 1. It is not necessary to include the abbreviation "PERFORM" in the title. [NOTE FROM THE EDITORS: Please rebut this comment, it is fine to keep the study abbreviation in the title] | As recommended by the editor, PERFORM will be kept in the title. | 1 |
| 2. The second page of the Background section could be shortened. Consider integrating some of the references into a slightly expanded first paragraph of the second Background section. | Thank you for your comment. While we appreciate the importance of conciseness, best practice according to the MRC Guidance on the Development and Evaluation of Complex Interventions and the TIDieR framework is to fully describe the intervention and its theoretical underpinnings. This ensures that the evidence base behind the intervention is transparent and that the intervention can be replicated by others. | 4,5 |
| 3. The information presented in Table 1 could be integrated into the main text; the table itself is not essential. | Thank you for this suggestion. The table has been removed and the information listed in the text itself. | 8/9 |
| 4. If possible, the results should also be presented disaggregated by patient sex. | Thank you for this suggestion. It was not an aim of the work to conduct ‘subgroup’ analyses of the qualitative data. We did not anticipate strong differences between men and women, so we did not divide the data into subgroups in this way for either gender or other demographics such as age. However, we remained vigilant during analyses of these kinds of differences and we didn’t identify these in the data. However, we did highlight some differences between site types that emerged, as this was relevant to future trials and implementation. | |

| | | |
|---|---|---|
| 5. The manuscript contains too many subheadings, particularly in the Discussion section; these could be reduced for improved clarity and flow. | Thank you for your comment. Sub-headings have been reduced or combined where possible, particularly in the Discussion section. We were not able to reduce further in the results, as this would remove clarity around themes, which is important in the reporting of qualitative results. The distinction between NPT and Other themes is needed to evidence how we used the theory in the analysis and provide a clear example for others wishing to use this approach. | Throughout |
| 6. The Discussion and Conclusion sections could be more concise. | We have made some edits to shorten this section, while retaining the space to reflect on the implications of the study findings. | 18-22 |
| 7. The reference numbering throughout the manuscript needs to be reviewed and corrected. Please do not include references that have not yet been published. | Thank you for your comment. References have been listed using automated referencing software. These have been checked, and all numbers correspond to the correct reference.<br><br>Of the two unpublished references cited in this manuscript, one has been accepted and is now in-press (18) and the other has been submitted alongside this manuscript as part of a dual submission to BMJ Open (17). | Throughout |
| 8. Please include the trial registration number. | Included | 2 |
| Reviewer 2 | | |
| It is overwhelming for the reader with too many aims , too many interpretations. Though the idea is very good but this needs clearly defined methodology. The author should add clarity by addressing primary objective first. | Thank you for your comments. We appreciate the importance of clarity and suggest that the structure as it stands, with clearly differentiated sections and headings, supports this. We respectfully disagree that there are too many aims. The aims are reflective of the | |

| | | |
|---|---|---|
| | purpose of a process evaluation. We have described the methods in full and followed the best practice in reporting process evaluations following the UK Medical Research Council Guidance on Process Evaluations and also the guidance for the Development and Evaluation of Complex Interventions. Regarding the number of interpretations, it is a qualitative study, and this requires interpretation of the specific themes found in the data. We are unclear what is meant by addressing the primary objectives first, as we have addressed the study aims/objectives and we don’t have a primary objective as such. Each aim/objective has equal weight. | |
